# Trends in hepatitis C virus seroprevalence and associated risk factors among msm in Pakistan: insights from a community-based study

**DOI:** 10.1038/s41598-024-63351-x

**Published:** 2024-07-17

**Authors:** Raza Tirmizi, Rimsha Munir, Nousheen Zaidi

**Affiliations:** 1 Dostana Male Health Society, Lahore, Pakistan; 2 Action Research Collective, Lahore, Pakistan; 3 Hormone Lab Lahore, Lahore, Pakistan; 4 Cancer Biology Lab, Institute of Microbiology and Molecular Genetics, University of the Punjab, Lahore, Pakistan; 5 Cancer Research Centre, University of the Punjab, Lahore, Pakistan

**Keywords:** HCV, Seroprevalence, MSM, HCV risk factors, Pakistan, Immunology, Diseases, Risk factors

## Abstract

Pakistan bears a substantial burden of hepatitis C virus (HCV) infection, with the second-highest prevalence globally. This community-based cross-sectional study, conducted from January to December 2022 in Punjab, Pakistan, investigates the seroprevalence of HCV among the men who have sex with men (MSM) population. The study identifies demographic and behavioral risk factors associated with HCV infection within this population group. Among the 501 participants, the study found an HCV seroprevalence of 14.86%. The association between demographic characteristics and seroprevalence is assessed by calculating the percentage of positive cases, revealing notable associations with age, education level, and self-identified sexual orientation. Furthermore, the study identified several behavioral risk factors positively associated with HCV seroprevalence, including sharing personal items such as razors and toothbrushes, histories of surgery, blood transfusion, dental procedures, intravenous drug use, and therapeutic injection histories. These risk factors were identified through structured interviews, and the prevalence of HCV seropositivity among the exposed groups was calculated accordingly. Interestingly, a lower HCV positivity rate was observed among self-reported HIV-positive individuals, contradicting previous research. The findings underscore the need for comprehensive, targeted prevention strategies such as risk factor awareness campaigns and educational programs tailored for the MSM population in Pakistan. Further research is warranted to validate these findings and better understand the complex interplay of factors contributing to HCV seroprevalence in this high-risk population.

## Introduction

Hepatitis C virus (HCV) infection, a leading cause of liver disease worldwide, manifests significant prevalence variation among different populations and locations^1^. According to the Global Hepatitis Report, approximately 71 million individuals were estimated to be living with chronic hepatitis C infection in 2015^2^. Multiple factors, including socioeconomic conditions^3–5^, healthcare accessibility^6^, and harm reduction programs^7^, inform this epidemiological diversity. Globally, two distinct demographic groups are mainly affected by HCV infections: younger individuals aged 20–40 years and older individuals over 50 years^8,9^.

HCV is transmitted predominantly via contact with contaminated blood through means such as contaminated surgical instruments, transfusion of contaminated blood or blood products, and sharing needles or syringes^10–12^. While the risk of transmission through sexual contact is relatively low, it is considerably higher among men who have sex with men (MSM)^13,14^. For MSM, especially those living with the human immunodeficiency virus (HIV), unprotected sex, notably when combined with *chemsex*, is recognized as a potential HCV transmission route^15,16^. Studies have consistently shown a higher prevalence of HCV infection in MSM compared to the general population^11,17,18^.

Notably, Pakistan bears a significant burden of HCV infection, with the second-highest prevalence globally^19^. Punjab, the most populous province in Pakistan, is known to have an exceptionally high prevalence of Hepatitis C Virus (HCV) infections. Approximately 10 million Pakistanis are estimated to be infected with HCV, with around 6.1% of these cases being active^20,21^. Despite this high prevalence, our understanding of behavioral determinants linked to HCV infection among MSM in Pakistan, a society where marginalized groups face formidable educational and healthcare barriers, remains limited.

In the Pakistani context, it is essential to note that only limited data on the seroprevalence of HCV among MSM is currently available. Furthermore, the existing data is derived from sporadic studies with small sample sizes, lacking detailed assessments of risk factors and sexual behavior patterns^20–24^. This highlights the critical need for a comprehensive study that addresses these limitations and provides a more thorough understanding of HCV seroprevalence and its associated factors among MSM in Pakistan.

By conducting a robust investigation encompassing a larger sample size and a detailed assessment of risk factors and sexual behaviors, our study aims to fill this knowledge gap and contribute to evidence-based interventions and strategies for HCV prevention and control among MSM in Pakistan. Our study overcame the challenges of researching disease prevalence in marginalized communities, such as Pakistan's MSM population, by collaborating with an organization called *Dostana Male Health Society*. Established in 2012 under the Societies Registration Act 1860, *Dostana* specializes in HIV prevention and community empowerment for at-risk and marginalized populations. Through our partnership with *Dostana,* we successfully engaged with the MSM community, addressing their unique barriers and conducting the study effectively. This collaborative approach aims to enhance our understanding of HCV infection dynamics among MSM in Pakistan, thus informing strategies to combat the HCV infection epidemic in this uniquely challenging social and healthcare setting.

## Methodology

### Research design and participant selection

This cross-sectional study determines the seroprevalence of the Hepatitis C Virus (HCV) among MSM in Pakistan. A total of 501 participants were included in this study.

### Recruitment of participants and inclusion and exclusion criteria

The inclusion criteria stipulated that participants must be MSM aged 15 years or older. Participants were recruited through the *Dostana Male Health Society,* a recognized society that addresses key populations' healthcare concerns. Due to Pakistan's societal and cultural barriers, engaging with individuals in these marginalized communities is challenging. As a solution, community members were educated and guided to gather data from the MSM population enlisted through *Dostana.*

### Data collection

The data collection process involved structured interviews. After the necessary training, data enumerators administered the structured questionnaires through one-on-one interviews. This method ensured that the process was consistent and comprehensive and aimed at minimizing potential bias or misinterpretation of the questions. The data were collected on demographic characteristics, common risk factors, and history of HIV.

### Ethical considerations

The study protocol is reviewed and approved by the Bioethics Committee of the Cancer Research Centre, University of the Punjab; the approval number is D/233-21/CRC. All participants provided informed written consent before participating in the study. The study is conducted following the WMA Declaration of Helsinki-Ethics principles for research involving human subjects.

### Serological testing

Blood samples are collected from participants for serological testing. Blood was drawn intravenously from all subjects, with 3 ml collected into a red top clot activator tube (containing no anticoagulants in Vacutainer) according to the guidelines of the National Committee for Clinical Laboratory Standards (H18-A4)^25^. To obtain serum, the clotted blood in tubes was centrifuged at 3000 rpm for 10 min. The isolated serum was then transferred to labeled tubes for further use. Following the manufacturer's instructions, the SD BIOLINE HCV Hepatitis C Virus Antibody Test kits were used to detect the presence of HCV antibodies in serum samples^26^. All components were brought to room temperature. After mixing the serum, the test device was opened, labeled, and loaded with 2–3 drops of serum. If no flow was observed within 30 s, Phosphate buffer saline (PBS) was added. The test was then initiated, and the results were interpreted within 15 min. A single line (C) indicated a negative result, two lines (C and T) were positive (requiring confirmation), and any absence of lines or presence of only the T line necessitated a retest. All tests are conducted in a certified laboratory following standard procedures.

### Statistical analysis

Descriptive statistics are used to summarize the demographic characteristics of the participants and the Prevalence of HCV. All analyses are performed using MS Excel. Seropositivity rates are determined by calculating the percentage of positive samples. The association between each risk factor and demographic characteristic with seroprevalence is assessed through the calculation of the percentage of positive cases.

## Results

### Demographic characteristics and HCV seroprevalence

**Figure 1:**
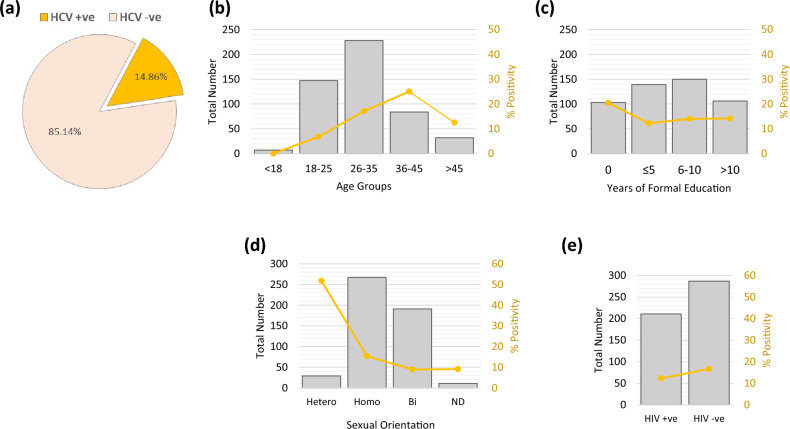
Prevalence of HCV seropositivity and associated demographic risk factors in Pakistani MSM. (**a**) A pie-chart displaying seroprevalence of HCV in our MSM population (n = 501). The total number of MSM participants in each (**b**) Age (**c**) Education (**d**) Sexual orientation and (**e**) Self-reported HIV status groups are plotted on *left y-axis* and represented as vertical *grey bars* and HCV seropositivity percentage is plotted on *right y-axis* and represented as a *yellow stacked line.*

For the presented work, 501 MSM participants were recruited to assess the seroprevalence of HCV. The data on the demographic characteristics were recorded through a structured interview by our survey enumerators. Our analysis of the demographics within the study group revealed a predominance of younger individuals, with those aged 18–35 making up 75.44% of the entire cohort. Additionally, it has been observed that a significant proportion of participants reported no formal education (20.55%) or had completed only five years (27.74%) or less. Our results indicate that HCV was positive in 14.86% of the study population (Fig. 1a).

### Association of HCV seroprevalence with selected demographic characteristics

We explored the relationships between demographic characteristics and HCV seroprevalence in our MSM population. We observed a noticeable trend of HCV positivity rates with varying age groups (Fig. 1b). From age 18, the HCV positivity increased steadily up to the age group of 36–45 years, which reported the highest HCV prevalence at 25%. Surprisingly, the rate decreased to 12.5% in respondents over 45.

We found an inverse relationship between years of formal education and the HCV positivity rate. The group with no formal education had the highest HCV positivity at 20.39%. However, HCV positivity remained relatively consistent as education levels increased (Fig. 1c).

Our data demonstrated variability in HCV positivity rates based on self-reported sexual orientation. Heterosexual respondents had a markedly high HCV positivity rate of 51.7%, significantly higher than in homo-, bi-, or undisclosed sexual orientation respondents, with HCV positivity rates of 15.3%, 8.9%, and 9.0%, respectively (Fig. 1d).

Further, we examined the interplay between HIV status and HCV positivity. Interestingly, a lower HCV positivity rate (12.3%) was observed among self-reported HIV-positive individuals compared to their HIV-negative counterparts (16.7%) (Fig. 1e).

### Association of HCV seroprevalence with common risk factors

**Figure 2:**
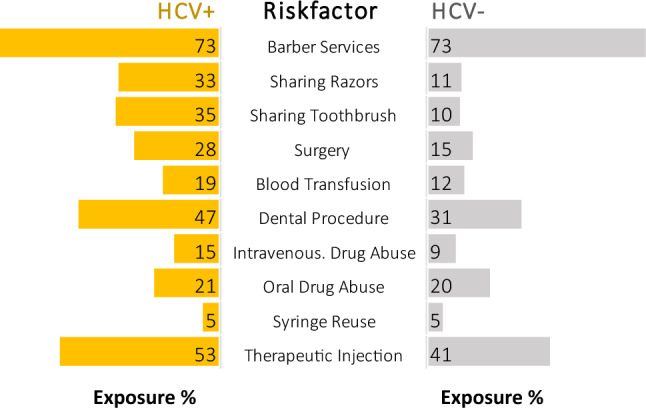
Exposure to selected behavioral risk factors in seropositive and seronegative MSM population. A *butterfly graph* displaying exposure percentage of different behavioral risk factors in seropositive and seronegative MSM population.

Next, we analyzed the relationship between HCV seropositivity and various commonly identified risk factors within the MSM population. The risk factors assessed were: utilization of barber services, sharing of razors or toothbrushes, surgical history, history of blood transfusion, dental procedures, intravenous and oral drug abuse, syringe reuse, and therapeutic injection history (Fig. 2).

The prevalence of each risk factor was compared between the HCV-positive and HCV-negative groups. Our data indicated a high percentage of individuals who use barber services in both the HCV-positive (73%) and HCV-negative groups (73%). Similarly, we observed comparable proportions of syringe reuse and oral drug abuse between the HCV-positive and HCV-negative groups.

However, several risk factors were markedly more prevalent in the HCV-positive group compared to the HCV-negative group. The sharing of razors was reported by 33% of HCV-positive individuals compared to 11% in the HCV-negative group. Similarly, sharing toothbrushes was significantly more prevalent in the HCV-positive group (35%) than in the HCV-negative group (10%).

Additionally, histories of surgery and blood transfusion were more common in the HCV-positive group (28% and 19%, respectively) compared to the HCV-negative group (15% and 12%, respectively). Dental procedure histories (47%), intravenous drug abuse (15%), and therapeutic injection histories (53%) were also higher in the HCV-positive group.

### Association of HCV seroprevalence with sexual risk behaviors

**Figure 3:**
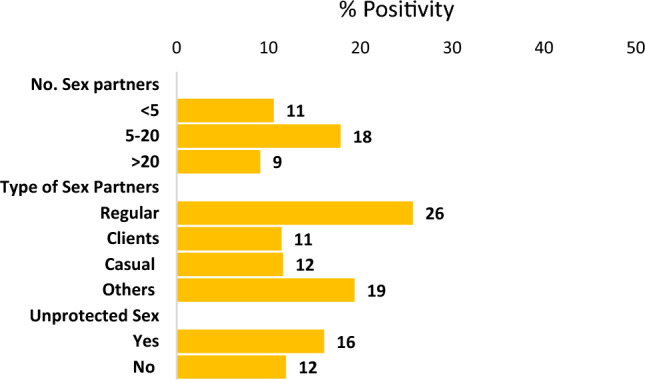
Association between HCV seropositivity and sexual risk behaviors in MSM. A *bar graph* displaying seropositivity percentage in MSM population engaging in different sexual risk behaviors.

Next, we investigated the association between sexual risk behaviors and HCV seroprevalence. The dataset included the percentage of HCV seropositivity for different sexual risk behaviors. Regarding the number of sex partners, individuals with less than 5 partners had an HCV positivity rate of 11%, while those with 5–20 partners exhibited a higher rate of 18%. Participants with more than 20 partners had a lower HCV positivity rate of 9% (Fig. 3).

Examining the type of sex partners, individuals involved with regular partners showed the highest HCV positivity rate at 26%. Those engaged with clients and casual partners had rates of 11% and 12%, respectively. Individuals involved with other types of partners had an HCV positivity rate of 19%.

Evaluating unprotected sex, participants who reported engaging in unprotected sexual activities had an HCV positivity rate of 16%, whereas those who practiced protected sex had a rate of 12%. These findings highlight the varying degrees of HCV positivity among individuals based on their sexual risk behaviors, emphasizing the importance of promoting safe sexual practices and tailored interventions to reduce HCV transmission.

## Discussion

In our study, we discovered a significantly high Hepatitis C Virus (HCV) seroprevalence of 14.86% among the men who have sex with men (MSM) population in Pakistan, a figure that substantially exceeds the global pooled seroprevalence of 3.4% among MSM^27^. This key finding underscores the critical epidemiological challenge of HCV within this demographic in Pakistan, revealing an urgent need for targeted health interventions. Additionally, we identified patterns of increased HCV positivity associated with certain age groups and reported higher rates of HCV positivity among self-identified heterosexual men within the MSM community, despite the limitations posed by a small sample size. These findings highlight the complex interplay of age, sexual behavior, and identity in influencing HCV risk, suggesting the need for nuanced public health strategies.

Upon delving deeper, we observed an age-associated increase in HCV positivity within the MSM population, peaking in individuals aged 36–45 years and subsequently declining among those over 45. This pattern could be attributed to age-related risk-taking behavior or a generational difference in awareness about HCV^28,29^, which warrants further investigation. Moreover, the increase in HCV positivity up to 36–45 years could be due to cumulative exposure to risk factors over time^30^. The decrease in positivity rates in respondents over 45 might be due to survivor bias, where those who are infected and do not receive treatment may not survive to older ages.

Our analysis revealed a notably high HCV positivity rate among self-identified heterosexual participants within the MSM group. This observation, while compelling, should be interpreted with caution due to the small sample size of this subgroup (n = 30), which limits the generalizability of this finding. With smaller sample sizes, a few cases of HCV positivity can significantly affect the overall percentage, leading to potentially inflated estimates of HCV prevalence in that group. Moreover, smaller sample sizes are generally associated with greater uncertainty and less statistical power, making it difficult to draw definitive conclusions. This subgroup's high HCV positivity rate could be attributed to undisclosed or stigmatized same-sex behaviors, but further research is necessary to substantiate this hypothesis. Future studies with larger sample sizes are warranted to validate this finding and to delve deeper into the potential underlying factors contributing to this elevated HCV positivity rate among self-identified heterosexual MSM.

Our study revealed a lower HCV positivity rate among HIV-positive individuals than their HIV-negative counterparts. This finding contrasts previous research, which typically reports increased rates of sexually transmitted HCV infection among the HIV-positive MSM population^31,32^. This discrepancy may be attributed to several factors. One possible explanation is that individuals living with HIV, due to their regular engagement with healthcare services, may have more frequent screening for HCV, better adherence to treatment, and more robust implementation of prevention strategies, which could contribute to a lower HCV prevalence in this group. However, it's also crucial to note that HIV seroprevalence was not directly tested in this study. Instead, we relied on self-reported HIV status, which could introduce bias and skew the results. Individuals may underreport their HIV status due to stigma or lack of awareness, leading to an underestimation of HCV positivity among the HIV-positive population. While these explanations are plausible, they remain speculative, and further research is needed to understand this counterintuitive finding fully.

Our study identified several risk factors that were positively associated with HCV seroprevalence among the MSM population in Pakistan. Sharing personal items like razors and toothbrushes was significantly more prevalent among HCV-positive individuals. Histories of surgery, blood transfusion, and dental procedures were more common among HCV-positive individuals. Intravenous drug use and therapeutic injection histories were also higher among HCV-positive individuals. These findings suggest that sharing personal hygiene items, histories of medical procedures, and practices associated with drug use may contribute to the higher prevalence of HCV infection among MSM individuals.

This study also examined the relationship between sexual risk behaviors and HCV seroprevalence. Our analysis involved assessing the percentage of HCV positivity for different sexual risk behaviors. Among individuals with varying numbers of sex partners, those with less than 5 partners had an HCV positivity rate of 11%. In contrast, individuals with 5–20 partners exhibited a higher HCV positivity rate of 18%, indicating an increased risk of HCV transmission with a higher number of partners. Interestingly, individuals with more than 20 partners had a lower HCV positivity rate of 9%, suggesting a potential decrease in risk beyond a certain threshold of sexual partners.

When considering the type of sex partners, individuals involved with regular partners had the highest HCV positivity rate at 26%, indicating a heightened risk of HCV transmission within stable sexual relationships. The higher HCV positivity rate among individuals involved with regular sex partners could be attributed to factors such as longer-term sexual relationships, increased likelihood of sharing needles or drug-related behaviors, or a higher prevalence of HCV infection within certain social networks.

Those engaged with clients and casual partners had comparatively lower seropositivity rates of 11% and 12%, respectively. This lower seroprevalence be influenced by factors such as commercial sex work practices, the use of protective measures during sexual encounters, or the lower prevalence of HCV infection within these specific populations.

In conclusion, our study sheds light on the diverse factors associated with HCV prevalence among MSM and underlines the need for comprehensive, targeted prevention strategies. However, limitations such as potential biases in self-reported data, the cross-sectional design's inability to establish causality, and limitations in sample representativeness should be acknowledged. Educational campaigns focusing on hygiene practices and safe disposal of contaminated materials are deemed essential. Additionally, the unexpectedly lower HCV positivity among self-reported HIV-positive individuals requires further investigation to understand the underlying factors and customize interventions accordingly. More research is necessary to verify our findings, particularly those related to age, education level, and sexual behavior. Our study contributes valuable insights that can guide the design of intervention programs, emphasizing education, safe practices, and increased healthcare engagement for this high-risk population.

## Data Availability

The datasets supporting the conclusions of this article are included within the article.
